# GLP-1 Analogs and DPP-4 Inhibitors in Type 2 Diabetes Therapy: Review of Head-to-Head Clinical Trials

**DOI:** 10.3389/fendo.2020.00178

**Published:** 2020-04-03

**Authors:** Matthew P. Gilbert, Richard E. Pratley

**Affiliations:** ^1^Division of Endocrinology and Diabetes, Department of Medicine, Larner College of Medicine at the University of Vermont, Burlington, VT, United States; ^2^AdventHealth Diabetes Institute, Translational Research Institute for Metabolism and Diabetes, Orlando, FL, United States

**Keywords:** incretin hormones, GLP-1 receptor agonists, DPP-4 inhibitors, cardiovascular outcomes trials, clinical trials, incretin biology, type 2 diabetes

## Abstract

The incretin hormones glucagon-like peptide-1 (GLP-1) and glucose-dependent insulinotropic polypeptide (GIP) are released from enteroendocrine cells in response to the presence of nutrients in the small intestines. These homones facilitate glucose regulation by stimulating insulin secretion in a glucose dependent manner while suppressing glucagon secretion. In patients with type 2 diabetes (T2DM), an impaired insulin response to GLP-1 and GIP contributes to hyperglycemia. Dipeptidyl peptidase-4 (DPP-4) inhibitors block the breakdown of GLP-1 and GIP to increase levels of the active hormones. In clinical trials, DPP-4 inhibitors have a modest impact on glycemic control. They are generally well-tolerated, weight neutral and do not increase the risk of hypoglycemia. GLP-1 receptor agonists (GLP-1 RA) are peptide derivatives of either exendin-4 or human GLP-1 designed to resist the activity of DPP-4 and therefore, have a prolonged half-life. In clinical trials, they have demonstrated superior efficacy to many oral antihyperglycemic drugs, improved weight loss and a low risk of hypoglycemia. However, GI adverse events, particularly nausea, vomiting, and diarrhea are seen. Both DPP-4 inhibitors and GLP-1 RAs have demonstrated safety in robust cardiovascular outcome trials, while several GLP-1 RAs have been shown to significantly reduce the risk of major adverse cardiovascular events in persons with T2DM with pre-existing cardiovascular disease (CVD). Several clinical trials have directly compared the efficacy and safety of DPP-4 inhibitors and GLP-1 RAs. These studies have generally demonstrated that the GLP-1 RA provided superior glycemic control and weight loss relative to the DPP-4 inhibitor. Both treatments were associated with a low and comparable incidence of hypoglycemia, but treatment with GLP-1 RAs were invariably associated with a higher incidence of GI adverse events. A few studies have evaluated switching patients from DPP-4 inhibitors to a GLP-1RA and, as expected, improved glycemic control and weight loss are seen following the switch. According to current clinical guidelines, GLP-1RA and DPP-4 inhibitors are both indicated for the glycemic management of patients with T2DM across the spectrum of disease. GLP-1RA may be preferred over DPP- 4 inhibitors for many patients because of the greater reductions in hemoglobin A1c and weight loss observed in the clinical trials. Among patients with preexisting CVD, GLP-1 receptor agonists with a proven cardiovascular benefit are indicated as add-on to metformin therapy.

## The Role of the Incretin System in Glucose Homeostasis

### Incretin Biology

Glucose homeostasis is dependent upon the complex interplay of multiple hormones including insulin, amylin, glucagon, and incretin hormones. The incretin hormones, principally glucagon-like peptide-1 (GLP-1) and glucose-dependent insulinotropic polypeptide (GIP), are released into the circulation in response to nutrients and facilitate insulin secretion in a glucose dependent manner. Collectively, the incretin hormones account for 50–70% of total post-prandial insulin secretion (1, 2). In patients with type 2 diabetes (T2DM), there is an impaired incretin effect that is likely multifactorial in nature (3, 4).

GLP-1 is secreted by L-cells found in the ileum, colon and rectum (5–7). Its physiologic role is to regulate plasma glucose in the post-prandial period through the facilitation of glucose stimulated insulin secretion, slowing of gastric emptying and suppression of glucagon secretion (8–15). In patients with diabetes, the insulin response to GLP-1 is blunted. This defect can be overcome with infusions that achieve supraphysiologic GLP-1 levels. The slowing of gastric emptying may be more important than insulin secretion in regulating post-prandial hyperglycemia by limiting the amount of post-prandial glucose presented to the beta cell (16).

GIP is released from K-cells found predominately in the duodenum and proximal gut in response to nutrients. Like GLP-1, GIP enhances insulin secretion from pancreatic beta cells in a glucose dependent manner, but it does not seem to suppress glucagon secretion in the same way. The response to GIP is also markedly impaired in patients with T2DM. Unlike GLP-1, supraphysiologic GIP infusions do not amplify the late phase insulin response to glucose in patients with T2DM (17, 18).

Native GLP-1 and GIP have limited pharmacologic value because of their short plasma half-life (1–7 min) (19). Both endogenous and exogenous GLP-1 and GIP are rapidly metabolized and inactivated by dipeptidyl peptidase-4 (DPP-4) (20) which is broadly expressed on cell surfaces and present in the circulation.

### Leveraging the Incretin Effect for the Treatment of T2DM

Because of the apparent lack of beta-cell responsiveness to GIP in patients with poorly controlled T2DM, most therapeutic strategies have focused on enhancing the activity of GLP-1. Two strategies have been employed to elevate and sustain GLP-1 mediated effects. The first relies on inhibition of DPP-4; this strategy effectively extends the half-life of endogenous GLP-1 and GIP, but is dependent on endogenous incretin hormone production. The second strategy is the use of GLP-1 receptor agonists (GLP-1RA) that are resistant to DPP-4 degradation. Treatment with GLP-1RAs can provide supraphysiologic and sustained stimulation of the GLP-1 receptor. While these two classes of antihyperglycemic medications work through similar pathways, their efficacy and side effect profiles differ, due to differences in the pharmacodynamic effect of DPP-4 inhibitors and GLP-1RAs with respect to enhancing GLP-1 activity.

### DPP-4 Inhibitors

DPP-4 inhibitors are low molecular-weight, orally available drugs that rapidly and specifically inhibit DPP-4 activity. DPP-4 is a ubiquitous enzyme present in the circulation and expressed on the surface of most cell types that has been found to inactivate GLP-1 and GIP. By preventing this, DPP-4 inhibitors enhance active GLP-1 and GIP levels by 2 to 3-fold following a meal (21). All approved DPP-4 inhibitors appear to have similar glycemic efficacy resulting in moderate (0.5–0.8%) reduction in HbA1c (22). Very few head-to-head trials have directly compared DPP-4 inhibitors. In an 18 week trial of in 800 patients with inadequately controlled T2DM on metformin, saxagliptin 5 mg daily vs. sitagliptin 100 mg showed similar reductions in hemoglobin A1c (HbA1c) (−0.52 vs. −0.26%) (23). Results from a meta-analysis of studies comparing sitagliptin with placebo or vildagliptin with placebo also showed similar clinical efficacy with respect to lowering of A1c (24). DPP-4 inhibitors are weight neutral due to the limited increase in GLP-1 activity (25–27). The incretin hormone GLP-1 has little effect on insulin secretion by pancreatic beta cells in the absence of elevated blood glucose derived from gut absorption. The risk of hypoglycemia with DPP-4 inhibitors is low given their GLP-1 mediated glucose dependent mechanism of action.

### GLP-1 Receptor Agonists

Two classes of GLP-1RAs have been developed based on the exendin-4 molecule and human GLP-1 (28, 29). All GLP-1RAs bind with specificity to the GLP-1 receptor and stimulate glucose dependent insulin release from the pancreatic beta cells (30). GLP-1RAs are described as short acting or long acting, based on their pharmacokinetic and pharmacodynamic properties. Short-acting GLP-1RAs have a half-life of 2–4 h thus necessitating once or twice daily administration (28, 29). Long-acting GLP-1RAs have a half-life >12 h (liraglutide), with others, such as albiglutide, dulaglutide, exenatide extended release, and semaglutide having a half-life as long as 14 days (31). These properties allow for once weekly administration of the longer-acting GLP-1RAs.

The pharmacodynamic effect of GLP-1RAs on glycemic control differs between short-acting and long-acting preparations. Short-acting GLP-1RAs primarily lower the post-prandial glucose response by slowing gastric emptying in addition to enhancing insulin secretion (29, 31). Long-acting GLP-1RAs lower the fasting blood glucose level by stimulating insulin secretion and reducing glucagon over an extended period of time. They appear to have less prominent effects on post-prandial glucose excursions, perhaps because the effects on gastric motility are decreased due to tachyphylaxis (29, 31). This difference in pharmacodynamics, as well as the differing half-lives, may contribute to the differences in efficacy seen in clinical trials.

Shyangdan et al. (32) performed a meta-analysis of 17 randomized trials comparing GLP-1RA (exenatide, liraglutide, albiglutide, taspoglutide, lixisenatide). When compared with placebo, all GLP-1RAs reduced HbA1c by ~1–1.2%. The risk of hypoglycemia is low with GLP-1RAs because of their glucose dependent mechanism of action. However, it is higher when GLP-1RAs are used in combination with sulfonylureas (33–37).

Generally, longer acting GLP-1RAs are more efficacious with respect to glycemic control than shorter acting GLP-1RAs. For example, in patients with T2DM, the once-weekly formulation of exenatide has been shown to provide greater reduction in HbA1c than the shorter acting exenatide twice-daily formulation. Both liraglutide and dulaglutide have also demonstrated better glycemic control over exenatide twice daily (38, 39). In more recent studies comparing long acting GLP-1RAs, once weekly semaglutide demonstrated superior glycemic efficacy and weight loss compared to exenatide extended release (SUSTAIN-3) or dulaglutide (SUSTAIN-7) (40, 41)

## Head-to-Head Studies OF DPP-4 Inhibitors vs. GLP-1 RAs

Several clinical trials have compared the clinical efficacy and safety profiles of GLP-1 RA and DPP-4 inhibitors in patients with T2DM (Table 1). In all the clinical trials between the two classes of medications, only sitagliptin has been studied in direct comparisons with a GLP-1RA. However, given the similar clinical efficacy and safety profiles amongst the available agents within the DPP-4 class of medications, the published head-to-head trials reviewed in this manuscript represent a good comparison of the two classes of medications (26, 35).

**Table 1:** Changes in HbA1c, FPG, PPG, and weight from the randomized, head-to-head trials comparing GLP-1RA vs. sitagliptin.

**GLP-1RA**	**Study**	**Duration (n)**	**GLP-1RA dose**	**Sitigliptin dose**	**Background medication**	**GLP1-RA vs. sitagliptin**			
						**Δ HbA1c**	**Δ FPG**	**Δ PPG**	**Δ Weight**
Exenatide	DeFronzo et al. (42)	2 weeks (61)	5 μg BID × 1 week then 10 μg BID × 1 week	100 mg/day	Metformin	N/A	N/A	−111.6 vs. −37.8 mg/dL (*p* < 0.0001)*	−0.8 vs. −0.3 kg (*p* < 0.006)^†^
	Berg et al. (43)	8 weeks (86)	10 μg BID	100 mg/day	Metformin or TZD	N/A	N/A	−108 vs. −45 mg/dL (*p* < 0.001)^†^	−1.37 vs. −0.89 kg (*p* < 0.05)^†^
	Bergenstal et al. (44)	26 weeks (514)	2 mg QW	100 mg/day	Metformin	−1.5 vs. −0.9% (*p* < 0.0001)*	−32.4 vs. −16.2 mg/dL (*p* < 0.0001)^†^	N/A	−2.3 vs. −0.8 kg (*p* < 0.0002)^†^
Liraglutide	Pratley et al. (45)	26 weeks (665)	1.8 mg/day	100 mg/day	Metformin (≥1,500 mg/day)	−1.5 vs. −0.9% (*p* < 0.001)*	−38.5 vs. −14.9 mg/dL (*p* < 0.0001)^†^	N/A	−3.38 vs. −0.96 kg (*p* < 0.001)^†^
			1.2 mg/day	100 mg/day	Metformin (≥1,500 mg/day)	−1.24 vs. −0.9% (*p* < 0.001)*	−33.6 vs. −14.9 mg/dL (*p* < 0.001)^†^	N/A	−2.86 vs. −0.96 kg (*p* < 0.001)^†^
Lixisenatide	Van Gaal et al. (46)	24 weeks (319)	20 μg/day	100 mg/day	Metformin	−0.7 vs. −0.7%*	−8.1 vs. −12.4 mg/dL (*p* < 0.3342)^†^	−60.3 vs. −25.9 mg/dL *(p* < 0.001)^†^	−2.51 vs. −1.17 kg (*p* < 0.0006)^†^
Taspoglutide	Bergenstal et al. (47)	24 weeks (666)	10 mg QW	100 mg/day	Metformin (≥1500 mg/day)	−1.23% vs. −0.89% (*p* < 0.001)*	−38.8 vs. −24.3 mg/dL (*p* < 0.001)^†^	N/A	−1.8 vs. −0.9 kg (*p* < 0.001)^†^
			20 mg QW	100 mg/day	Metformin (≥1,500 mg/day)	−1.30 vs. −0.89% (*p* < 0.001)*	−42.1 vs. −24.3 mg/dL (*p* < 0.001)^†^	N/A	−2.6 vs. −0.9 kg (*p* < 0.001)^†^
Albiglutide	Ahren et al. (48)	104 weeks (1049)	50 mg QW	100 mg/day	Metformin	−0.63 vs. −0.28% (*p* < 0.0001)*	−28.0 vs. −16 mg/dL (*p* < 0.0001)^†^	N/A	−1.21 vs. −0.86 kg^†^
	Leiter et al. (49)	26 weeks (507)	50 mg QW	Dose based on eGFR	Metformin, TZD, sulfonylurea (alone or any combination)	−0.83 vs. −0.52% (*p* < 0.003)*	−25.5 vs. −3.96 mg/dL (*p* < 0.0001)^†^	N/A	−0.79 vs. −0.19 kg (*p* < 0.05)^†^
Dulaglutide	Nauck et al. (50)	52 weeks (1098)	1.5 mg QW	100 mg/day	Metformin	−1.10 vs. −0.39% (*p* < 0.001)*	−42 vs. −20 mg/dL (*p* < 0.001)^†^	N/A	−3.03 vs. −1.53 kg (*p* < 0.001)^†^
			0.75 mg QW	100 mg/day	Metformin	−0.87 vs. −0.39% (*p* < 0.001)*	−33 vs. −20 mg/dL (*p* < 0.001)^†^	N/A	−2.26 vs. −1.53 kg (*p* < 0.001)^†^
	Weinstock et al. (51)	104 weeks (1098)	1.5 mg QW	100 mg/day	Metformin	−0.99 vs. −0.32% (*p* < 0.001)*	−36 vs. −9.0 mg/dL (*p* < 0.001)^†^	N/A	−2.88 vs. −1.75 kg (*p* < 0.05)^†^
			0.75 mg QW	100 mg/day	Metformin	−0.71 vs. −0.32% (*p* < 0.001)*	−25.2 vs. −9.0 mg/dL (*p* < 0.001)^†^	N/A	−2.39 vs. −1.75 kg^†^
Semaglutide	Ahren et al. (52)	56 weeks (1231)	0.5 mg QW	100 mg/day	Metformin, TZD, or both	−1.3 vs. −0.5% (*p* < 0.0001)*	−37.8 vs. −19.8 mg/dL (*p* < 0.0001)^†^	N/A	−4.3 vs. −1.9 kg (*p* < 0.0001)^†^
			1.0 mg QW	100 mg/day	Metformin, TZD, or both	−1.6 vs. −0.5% (*p* < 0.0001)*	−46.8 vs. −19.8 mg/dL (*p* < 0.0001)^†^	N/A	−6.1 vs. −1.9 kg (*p* < 0.0001)^†^
	Seino et al. (53)	30 weeks (308); Japanese	0.5 mg QW	100 mg/day	OAD monotherapy	−1.9 vs. −0.7% (*p* < 0.0001)^†^	−50.4 vs. −23.4 mg/dL (*p* < 0.0001)^†^	N/A	−2.2 vs. −0.0 kg (*p* < 0.0001)^†^
			1.0 mg QW	100 mg/day	OAD monotherapy	−2.2 vs. −0.7% (*p* < 0.0001)^†^	−59.4 vs. −23.4 mg/dL (*p* < 0.0001)^†^	N/A	−3.9 vs. −0.0 kg (*p* < 0.0001)^†^
	Rosenstock et al. (54)	78 weeks (1864) NOTE: Data shown is for baseline to 26 weeks	Oral semaglutide 3 mg/day	100 mg/day	Metformin with or without a sulfonylurea	−0.6 vs. −0.8% (*p* < 0.09)*	−13.6 vs. −15.4 mg/dL (*p* < 0.50)^†^	N/A	−1.2 vs. −0.6 kg (*p* < 0.61)^†^
			Oral semaglutide 7 mg/day	100 mg/day	Metformin with or without a sulfonylurea	−1.0 vs. −0.8% (*p* < 0.001)*	−21.3 vs. −15.4 mg/dL (*p* < 0.04)^†^	N/A	−2.2 vs. −0.6 kg (*p* < 0.001)^†^
			Oral semaglutide 14 mg/day	100 mg/day	Metformin with or without a sulfonylurea	−1.3 vs. −0.8% (*p* < 0.001)*	−30.5 vs. −15.4 mg/dL (*p* < 0.001)^†^	N/A	−3.1 vs. −0.6 kg (*p* < 0.001)^†^

*GLP-1RA, glucagon-like peptide-1 receptor agonist; HbA1c, glycosylated hemoglobin; FPG, fasting plasma glucose; PPG, postprandial glucose; TZD, thiazolidinedione; N/A, data not available; BID, twice a day; QW, once weekly; OAD, oral antidiabetic drug*.

* *Primary study endpoint*;

† *Secondary study endpoint*.

### Exenatide BID vs. Sitagliptin

Comparisons of exenatide twice daily with sitagliptin include two short crossover clinical studies. DeFronzo et al. (42) conducted a double-blind, randomized, crossover, multi-center study in patients with T2DM treated with metformin. This study evaluated the effects of exenatide (5 and 10 mcg) vs. sitagliptin (100 mg daily) on a number of clinical outcomes including post-prandial glucose, gastric emptying, and secretion of insulin and glucagon. After 2 weeks, patients crossed over to the alternate therapy. Exenatide demonstrated a greater reduction in 2-h post-prandial glucose, compared to sitagliptin. The authors concluded that the impact on post-prandial glucose was related to the greater effect of exenatide on GLP-1 receptor stimulation. This increased effect on the GLP-1 receptor improved post-prandial insulin secretion while reducing glucagon secretion. In addition, the study demonstrated that treatment with exenatide slowed gastric emptying and reduced caloric intake when compared to sitagliptin (42).

Berg et al. (43) conducted an 8-week, randomized, active-comparator, crossover study comparing the effects of exenatide twice daily vs. sitagliptin on glucose profiles in patients with T2DM. Eighty-six subjects received either exenatide 10 mcg subcutaneous twice daily or sitagliptin 100 mg orally daily for 4 weeks each. The primary outcome was time averaged glucose during the 24-h inpatient visits. Both treatments decreased average 24-h glucose and 2-h post-prandial glucose with and increased the amount of time spent with glucose values between 70 and 140 mg/dL. However, exenatide had a statistically significant greater effect in these clinical outcomes compared to sitagliptin. Adverse events were described as mild to moderate and were mostly gastrointestinal (GI) in patients treated with exenatide. This trial had several limitations, including the lack of a washout period between the two treatments. However, given the short half-lives of sitagliptin and exenatide, the impact of the lack of a washout period was likely not clinically relevant (55, 56).

### Lixisenatide vs. Sitagliptin

Lixisenatide is a once daily, exenatide-based short-acting GLP-1RA for the treatment of T2DM. Van Gaal et al. (46) conducted a 24-week, randomized, active-controlled, trial that compare the clinical efficacy of lixisenatide vs. sitagliptin in obese (BMI ≥30 kg/m^2^) patients <50 years of age with T2DM sub optimally controlled on metformin monotherapy. Patients were randomized to lixisenatide 20 mcg daily injection or oral sitagliptin 100 mg daily. Least squares mean change from baseline for HbA1c were similar for lixisenatide and sitagliptin (−0.7 and −0.7%, respectively). A similar proportion of patients in each group achieved a HbA1c of <7.0% (40.7% for lixisenatide vs. 40.0% for sitagliptin). Lixisenatide therapy was associated with greater reductions in body weight vs. sitagliptin by end of study (least squares mean change of −2.5 kg for lixisenatide vs. −1.2 kg for sitagliptin). Lixisenatide-treated patients also showed significantly greater reductions in 2 h post-prandial glucose (least squares mean change from baseline was −60.3 mg/dL for lixisenatide and −25.9 mg/dL for sitagliptin). There were no significant differences between groups in change in fasting plasma glucose levels. GI disorders were slightly more frequent with lixisenatide than sitagliptin. Nausea was the most frequently reported GI adverse event in the patients treated with lixisenatide.

### Liraglutide vs. Sitagliptin

Liraglutide is a long-acting GLP-1RA that has been modified to non-covalently bind to serum albumin through addition of an acyl side chain, resulting in slower degradation (half-life 11–15 h) and allowing for once-daily, subcutaneous dosing (57).

Pratley et al. (45) conducted an open-label, active-comparator trial to evaluate the clinical efficacy and safety profile of liraglutide vs. sitagliptin in individuals with T2DM who were not well-controlled with metformin monotherapy. Participants were randomized to receive 26 weeks of treatment with either 1.2 or 1.8 mg subcutaneous liraglutide once daily or 100 mg of sitagliptin once daily.

HbA1c reductions, from a baseline 8.5%, were larger with 1.2 and 1.8 mg of liraglutide vs. sitagliptin. At the completion of the trail, the mean decreases in HbA1c from baseline were −1.5% for 1.8 mg of liraglutide, −1.24% for 1.2 mg of liraglutide, and 0.9% for sitagliptin. Mean weight loss was greater with liraglutide than sitagliptin (−3.38 kg for 1.8 mg of liraglutide; −2.86 kg for 1.2 mg of liraglutide; −0.96 kg for sitagliptin).

With regard to safety, more adverse events were reported with liraglutide than with sitagliptin. Nausea was more common in patients treated with liraglutide (1.2 and 1.8 mg/day) compared to sitagliptin. The most common adverse events were GI symptoms, especially with liraglutide. Hypoglycemia was infrequent and comparable between groups.

### Exenatide Once Weekly vs. Sitagliptin

Bergenstal et al. (44) conducted a 26-week randomized, double-blind, trial that assessed the safety and efficacy of once-weekly exenatide (2 mg, *n* = 170) vs. sitagliptin (100 mg daily; *n* = 172) or pioglitazone (45 mg daily; *n* = 172) in patients with T2DM treated with metformin. Those patients treated with exenatide once weekly had a significantly greater reduction in HbA1c when compared to treatment with pioglitazone by week 4, or sitagliptin by week 6. The percentage of patients achieving a fasting blood glucose target of ≤126 mg/dL with exenatide once-weekly (60%) was significantly greater than with sitagliptin (35%) and was similar to pioglitazone (52%). At week 26, weight loss with exenatide once weekly (−2.3 kg) was significantly greater than with sitagliptin (−0.8 kg) or pioglitazone (+2.8 kg). A total of 28% of patients receiving exenatide had weight loss ≥5% compared with 10% of those on sitagliptin and 2% of those on pioglitazone. Patients treated with exenatide once weekly had significantly greater reduction in systolic blood compared to those on sitagliptin. The most common adverse events were nausea and diarrhea in patients treated with sitagliptin and exenatide once weekly. Upper respiratory tract infection and peripheral edema were most common in patients treated with pioglitazone. Minor hypoglycemic events were uncommon and rates were similar between all three treatment groups.

### Albiglutide vs. Sitagliptin

Albiglutide is a once weekly, long-acting GLP-1RA. The chemical structure allows an extended half-life and once weekly dosing (58, 59). Although albiglutide was approved in several countries, it is not currently being marketed.

Ahren et al. (48) conducted a 104-week randomized controlled trial assessing the efficacy and safety of albiglutide compared with placebo, sitagliptin, and glimepiride in patients with T2DM on metformin monotherapy. Eligible patients were randomly assigned to receive 1 of 4 treatments in addition to their background metformin: Albiglutide 30 mg, sitagliptin 100 mg, glimepiride 2 mg, or placebo.

Changes in HbA1c from baseline were greatest for albiglutide (−0.63%). Changes in HbA1c were also see in participants treated with sitagliptin (−0.28%), and glimepiride (−0.36%). Participants in the placebo arm had a 0.27% increase in HbA1c from baseline to week 104. This study concluded that treatment with albiglutide was superior to sitagliptin and glimepiride in addition to placebo (48). At week 104, treatment with albiglutide (−1.21 kg), placebo (−1.0 kg), and sitagliptin (−0.86 kg) resulted in weight loss. In contrast, patients treated with glimepiride had weight gain (+1.17 kg).

The proportion of GI adverse events were similar in the albiglutide and placebo groups. The most common GI adverse event was diarrhea in albiglutide group while constipation was most commonly seen in the placebo group. At the conclusion of the 104 week study, the proportion of patients who experienced nausea was similar between treatment groups. Rates of symptomatic hypoglycemia were similar with albiglutide (3%) compared to placebo (4%) and sitagliptin (1.7%). Patients treated with glimepiride experienced more symptomatic hyperglycemia (17.9%). No severe hypoglycemic events were seen in this clinical trial.

Another trial comparing albiglutide and sitagliptin was conducted by Leiter et al. (49). This randomized, phase three study looked at the efficacy and safety of once weekly albiglutide vs. sitagliptin in patients with T2DM and renal impairment. Renal impairment was categorized as mild, moderate, or severe as assessed by estimated glomerular filtration rate (49). HbA1c change from baseline at week 26 was greater for albiglutide vs. sitagliptin (−0.83 vs. −0.52%). Decreases in HbA1c, fasting plasma glucose, and weight were seen through week 52. The safety profiles were similar between groups and most adverse events were described as mild to moderate. The authors concluded that treatment with once weekly albiglutide in renally impaired patients showed statistically superior glycemic control with a similar safety and tolerability profile when compared with sitagliptin (49).

### Dulaglutide vs. Sitagliptin

Dulaglutide is a long-acting human GLP-1RA with a larger size molecule that slows absorption and reduces renal clearance (50). These features resulted in a prolonged half-life of ~5–6 days, allowing for once weekly administration (60).

Nauck et al. (50) conducted a multi-center, double-blind, parallel-arm, randomized controlled trial comparing the efficacy and safety of two doses of once weekly dulaglutide (0.75 and 1.5 mg) to sitagliptin in patients with suboptimally controlled T2DM on metformin monotherapy (AWARD-5 clinical trial). The mean HbA1c changes from baseline to week 52 were −1.1% for dulaglutide 1.5 mg, −0.87% for dulaglutide 0.75 mg, and −0.39% for sitagliptin (50). The mean changes in body weight from baseline to 52 weeks were significantly greater for dulaglutide 1.5 mg (−3.03 kg) and dulaglutide 0.75 mg (−2.6 kg) compared with sitagliptin (−1.53 kg). A decrease in systolic blood pressure was observed in all the active treatment arms, with the greatest reductions observed during the first 3–6 months of treatment (50). An increase of 2 to 3 heartbeats per minute (bpm) was observed with both dulaglutide doses. No increase in heart rate was seen in the sitagliptin or placebo arms.

The incidence of GI adverse events was significantly higher with dulaglutide compared with sitagliptin and placebo. The incidence of nausea, diarrhea, and vomiting with dulaglutide 1.5 mg and dulaglutide 0.7 mg was highest within the first 2 weeks of treatment and subsequently declined between 8 and 52 weeks of treatment (50). The incidence of hypoglycemia at 52 weeks was 10.2% for dulaglutide 1.5 mg, 5.3% for dulaglutide 0.75 mg, and 4.8% for sitagliptin. Changes in HbA1c at the conclusion of the study were −0.99, −0.71, and −0.32% for dulaglutide 1.5 mg, dulaglutide 0.75 mg and sitagliptin, respectively (51).

### Semaglutide vs. Sitagliptin

Semaglutide is a GLP-1RA with 94% homology to native GLP-1 (61). It is similar in structure to liraglutide, but less susceptible to degradation by DPP-4 (61). These structural modifications improve binding to albumin and result in a half-life of ~7 days. This allows for once weekly administration of subcutaneous semaglutide (61).

SUSTAIN 2 was a clinical trial that compared the efficacy and safety of semaglutide compared with sitagliptin as add on treatment in patients with T2DM on metformin, thiazolidinedione (TZD), or both (52). The trial was a 56-week, randomized, double blind, active-controlled, parallel-group trial that was conducted at 128 sites around the world.

At week 56, mean HbA1c had significantly decreased with semaglutide 0.5 mg and 1.0 mg by 1.3 and 1.6%, respectively, vs. 0.5% with sitagliptin. Mean body weight was reduced by 4.3 kg with semaglutide 0.5 mg and 6.1 kg with semaglutide 1.0 mg vs. a reduction of 1.9 kg with sitagliptin.

The proportion of participants reporting adverse events and serious adverse events were similar between groups. Seventy-three participants (18%) in the semaglutide 0.5 mg group and 72 participants (18%) in the semaglutide 1.0 mg group reported nausea vs. 30 of participants (7%) in the sitagliptin group. Nausea diminished over time in the semaglutide treatment group. The GI adverse events in the semaglutide groups were mild or moderate in severity. There were 3 events of acute pancreatitis in the semaglutide 0.5 mg group and one event of chronic pancreatitis in the semaglutide 1.0 mg group. No cases of pancreatitis were reported in the sitagliptin group.

Seino et al. (53) conducted a randomized, open-label, parallel-group, active-controlled trial to evaluate the safety and efficacy of monotherapy with semaglutide once-weekly vs. sitagliptin daily in Japanese patients with T2DM. The mean HbA1c decreased by 1.9 and 2.2% with semaglutide 0.5 and 1.0 mg, respectively, vs. 0.7% with sitagliptin. Body weight was reduced by 2.2 and 3.9 kg with semaglutide 0.5 and 1.0 mg, respectively. More adverse events were reported with semaglutide than sitagliptin.

### Oral Semaglutide vs. Sitagliptin

Up until recently, GLP-1RA could only be administered by subcutaneous injection. Rapid enzymatic-induced proteolytic degradation in the stomach leads to poor bioavailability of oral peptide medications (62). This leads to decreased absorption in the gastrointestinal tract and decreased clinical efficacy. An oral formulation of semaglutide has been made clinically possible through the combination of the drug with an “absorption enhancer” called SNAC (62). Clinical trials have demonstrated that once daily oral semaglutide improves HbA1c with a clinically significant reduction in body weight when compared to placebo (63).

PIONEER 3 was a 78-week, randomized, active-controlled, multi-center clinical trial. The trial compared the efficacy and safety of once-daily oral semaglutide vs. sitagliptin 100 mg in patients with T2DM on to metformin with or without a sulfonylurea (54).

Approximately 1,900 patients were randomized to receive semaglutide 3, 7, or 14 mg/day or sitagliptin 100 mg daily. Half of the patients in each treatment arm were also treaded with a sulfonylurea in addition to a stable dose of metformin (54).

The mean HbA1c changes from baseline with semaglutide 3 mg/day was −0.6%. For patients treated with semaglutide 7 and 14 mg/day there was a reduction in HbA1c of 1, and 1.3% over the 26 week trial. A reduction of 0.8% was observed in the patients treated with sitagliptin. The 7 and 14 mg/day semaglutide doses were shown to be statistically superior to sitagliptin in reducing HbA1c (54). The mean changes from baseline in body weight were −1.2, −2.2, and −3.1 kg for semaglutide, 3, 7, and 14 mg/day, respectively, and −0.6 kg for sitagliptin (54). The weight loss observed with semaglutide 7 and 14 mg/day was statistically significant when compared to sitagliptin. In addition, glycemic control was significantly greater with semaglutide 7 and 14 mg/day when compared to sitagliptin. The most frequently reported adverse events for oral semaglutide were GI disorders including nausea, emesis and diarrhea. Similar to the injectable forms of GLP-1RA, treatment oral semaglutide did not lead to an increased risk of pancreatitis or pancreatic cancer compared to placebo (54).

Pioneer 7 was a 52-week, randomized clinical trial enrolling patients with T2DM from 81 sites in 10 countries. The purpose of the trial was to compare the efficacy and safety of flexible dose adjustments of oral semaglutide compared to sitagliptin (64). Therefore, in order to approximate the individualization seen in “real world” clinical practice, oral semaglutide dose could be adjusted on the basis of glycemic control and tolerability criteria that were prespecified in the protocol. A total of 504 patients were randomized with a mean baseline HbA1c of 8.3%. A greater proportion of participants achieved a HbA1c of <7% with oral semaglutide (58%) than with sitagliptin (25%). At 52 weeks, estimated mean change in body weight was −2.6 kg for the semaglutide group and −0.7 kg for the sitagliptin group. Adverse events occurred in 78% of patients in the oral semaglutide group vs. 69% of patients in the sitagliptin group (64). Nausea was the most common adverse event with oral semaglutide and was reported in 21% of participants. The authors concluded that oral semaglutide, with dose adjustment based on efficacy and tolerability, provided superior glycemic control and weight loss compared with sitagliptin (64).

## Summary of DPP-4 Inhibitor vs. GLP-1 RA Head-to-Head Trials

In summary, these head-to-head clinical trials between GLP-1RA and DPP-4 inhibitors showed that the difference between the drugs lies in the ability of the GLP-1RA to consistently improve glycemic control and decrease weight (43). DPP-4 inhibitors increase active GLP-1 concentrations by 2 or 3 times the concentration at baseline. However, the stimulation of GLP-1 receptor activity with GLP-1RA is several times higher than with DPP-4 inhibitors (65, 66). The long half-life of GLP-1RA, particularly those products administered once-daily or once-weekly, may also contribute to increased efficacy (67).

## Switch Trials

A small number of clinical trials have evaluated switching patients from a DPP-4 inhibitor (sitagliptin) to a GLP-1RA. Wysham et al. (68) conducted a 26-week, open-label, uncontrolled follow-up assessment to evaluate the clinical efficacy and safety profile of switching from sitagliptin or pioglitazone to exenatide once-weekly. In this study, patients originally enrolled in the 26-week DURATION-2 study who had been randomized to sitagliptin or pioglitazone were switched to exenatide; patients who had been randomized to once-weekly exenatide continued the medication. Patients who continued treatment with exenatide maintained the significant improvements in HbA1c (−1.6%), fasting plasma glucose (−32 mg/dL), and weight (−1.8 kg) from baseline. Patients switched from sitagliptin to exenatide showed statistically significant improvements in HbA1c (−0.3%), fasting plasma glucose (−12.6 mg/dL) and weight (−1.1 kg). Exenatide was well-tolerated and adverse events were reported as mild or moderate. Nausea was the most frequent adverse event reported in patients treated with exenatide. There were no major hypoglycemic events observed. The authors concluded that patients who switched to exenatide from sitagliptin had improved glycemic control, with the addition of increased weight loss (68).

Pratley et al. (69) conducted a randomized, clinical trial to assess the efficacy and safety profile of switching from sitagliptin to liraglutide. Patients receiving metformin treatment for their T2DM were randomized to receive either liraglutide (1.2 or 1.8 mg/day) or sitagliptin (100 mg/day). After 52 weeks, those patients in the sitagliptin group were randomly assigned to liraglutide; all patients were treated for another 26 weeks. The authors reported that at the conclusion of the study there was an additional decrease in HbA1c of −0.2% after switching to liraglutide 1.2 mg/day. Switching to liraglutide was also associated with a reduction in fasting plasma glucose and weight. In addition, the authors noted an increase in the number of patients reaching HbA1c levels <7%. After switching, mild to moderate nausea occurred in 21% of patients, while rates of hypoglycemia remained low (3–4% of participants) (69). The authors concluded that glycemic control and weight improved after switching from sitagliptin to liraglutide with a transient increase in mild to moderate GI adverse events (69).

Bailey et al. (70) conducted a randomized, multi-centered, double-blind, double-dummy, active-controlled trial in attempt to confirm superiority of glycemic control when switching from sitagliptin to liraglutide vs. continuing sitagliptin. Subjects with inadequate glycemic control of T2DM on sitagliptin 100 mg/day and metformin ≥1,500 mg/day were randomized to either switch to liraglutide 1.8 mg/day or continue their sitagliptin; both groups continued the metformin regimen. There was greater reduction in mean HbA1c achieved with liraglutide 1.8 mg/day than with continued sitagliptin (−1.14 vs. −0.54%) and body weight was reduced more with liraglutide than with sitagliptin (−3.31 vs. −1.64 kg). However, nausea was more common with liraglutide (21.8%) than with continued sitagliptin (7.8%) and 3 subjects (1.5%) taking sitagliptin reported a confirmed hypoglycemic episode. The authors concluded that subjects inadequately controlled with sitagliptin in combination with metformin who switched to liraglutide 1.8 mg/day in combination with metformin had a clinically relevant reduction in HbA1c and body weight without significant adverse events (70).

## Add-on Trials

As both DPP-4 inhibitors and GLP-1RA work through enhancing GLP-1 activity, one might predict that combining the two classes would not be ideal. Nevertheless, Violante et al. (71) conducted a 20-week, randomized trial to evaluate the impact of treatment with the short-acting GLP-1RA exenatide after treatment with sitagliptin. Patients were randomized to treatment to one of two groups. The SWITCH group switched therapy from a combination of sitagliptin and metformin the exenatide and metformin. The ADD group had exenatide added to existing treatment with sitagliptin and metformin. Surprisingly, in this study of patients with T2DM inadequately controlled with sitagliptin and metformin, the primary non-inferiority objective of SWITCH therapy to ADD was not supported (71). There were no between-group differences in changes in body weight, lipid profile or blood pressure. Patients in both treatment groups had a lower incidence of nausea and emesis. The authors noted that the incidence of nausea and emesis were lower than those previously reported in other clinical trials of twice daily exenatide (24). The results of this trial suggest that adding a GLP-1RA to sitagliptin and metformin provides better glycemic control without a significant increase in adverse events. The results of this study supports current clinical practice of adding another oral anti-hyperglycemic medication is better than switching oral anti-hyperglycemic medications. Of note, however, is that the higher costs of these two classes of medications must also be considered in addition to the improved glycemic control.

Nauck et al. (72) conducted a small clinical trial to evaluate the addition of sitagliptin to pre-existing therapy with liraglutide. The study investigated changes glycemic excursions after a mixed meal test. Sixteen (16) patients with T2DM treated with metformin and liraglutide (1.2 mg/day for >2 weeks) were studied after two separate overnight fasts. The morning after the fast, patients received, in randomized order, sitagliptin 100 mg or placebo 60 min before a standard mixed meal test. Glucose excursions was the primary endpoint; insulin levels, C-peptide, glucagon, GLP-1 and GIP (total and intact) were also measured. Meal-induced responses of intact GLP-1 and GIP were augmented by the sitagliptin treatment by 78 and 90%, respectively (*p* < 0.001), when compared to placebo. Treatment with sitagliptin did not affect concentrations of C-peptide, insulin or glucagon. Glucose concentrations were also not affected by sitagliptin treatment. The authors concluded that treatment with a single dose of sitagliptin, in patients already treated with the GLP-1RA liraglutide, resulted in elevations in intact GLP-1 and GIP plasma concentrations after a mixed meal test, but without changes in insulin, C-peptide, glucagon and glucose concentrations. The authors noted several limitations, most importantly that the study only included a single dose of sitagliptin. Thus, one cannot draw conclusions regarding the possible clinical impact of longer-term treatment. In addition, the authors noted that the results should not be generalized to other GLP-1RA with different pharmacokinetic profiles (72).

## Cardiovascular Outcomes

### DPP-4 Inhibitors

Five trials enrolling almost 50,000 patients have evaluated the cardiovascular safety of DPP-4 inhibitors. All five trials met the primary objective of excluding an increased risk of cardiovascular disease in patients treated with DPP-4 inhibitors, however, none were associated with any cardiovascular benefit (73–75). The SAVOR-TIMI 53 (Saxagliptin Assessment of Vascular Outcomes Recorded in Patients with Diabetes Mellitus-Thrombolysis in Myocardial Infarction) trial with saxagliptin suggested an increased risk for incident heart failure (73). This finding coupled with a trend in the increased incidence of heart failure noted in the EXAMINE (Examination of Cardiovascular Outcomes with Alogliptin vs. Standard of Care) trial with alogliptin, prompted the FDA to issue a warning regarding heart failure risk, especially in patients with previously diagnosed cardiovascular and renal disease (76). However, overall the DPP-4 inhibitor class of medications has established cardiovascular safety.

### GLP-1 Receptor Agonists

The cardiovascular safety of GLP-1RA have been assessed in seven clinical trials enrolling 60,090 patients (77–80). The ELIXA trial enrolled patients with a history of recent acute coronary syndrome. The results were “cardiovascular neutral” confirmed, this confirming the inferiority of lixisenatide with respect to a four-point MACE (major adverse cardiovascular events) but did not show beneficial effects on cardiovascular outcomes (77). The second trial to be released was the LEADER trial, which not only demonstrated cardiovascular non-inferiority, but also showed the statistical superiority, of once-daily treatment with liraglutide. The reduction and 3-point MACE with liraglutide was driven by a significant reduction in cardiovascular death. All-cause mortality was also significantly reduced with liraglutide (78). There was no reduction in hospitalization for heart failure in the LEADER trial. SUSTAIN-6 confirmed the non-inferiority of once weekly treatment with 0.5 or 1 mg of once weekly GLP-1RA semaglutide. The favorable effect on 3-point MACE was driven by a significant decrease in non-fatal stroke. There was no trend for cardiovascular death or all-cause mortality (79). The fourth trial, EXSCEL was conducted in patients with T2DM with or without previous cardiovascular disease. The trial confirmed the non-inferiority, but not superiority, of once weekly treatment with 2 mg of the long-acting extended release exenatide (80). The fifth trial, REWIND showed that a weekly injection of 1.5 mg of dulaglutide reduced the risk of cardiovascular outcomes compared with placebo, with benefit starting within the first year of treatment (81). The sixth trial, PIONEER 6 published in 2019 and was a pre-registration cardiovascular safety trial for oral semaglutide. Patients were randomly assigned to either 14 mg once daily oral semaglutide or placebo and were stratified to established cardiovascular disease or chronic kidney disease or the presence of cardiovascular risk factors only. The primary composite outcome occurred in 3.8% of patients receiving oral semaglutide compared to 4.8% receiving placebo, which was not significant (HR 0.79, 0.57–1.11) (82).

The differences in cardiovascular outcomes between the various GLP-1RA currently available for the treatment of patients with T2DM may be the result of differences in study design or patient populations. The results may reflect differences in pharmacokinetic and pharmacodynamic properties, trial design or drug differences. One potential explanation includes the amount of structural similarity to human GLP-1 (76).

## Safety and Tolerability of GLP-1RAs and DPP-4 Inhibitors

The DPP-4 inhibitors have been well-tolerated in both short- and long-term studies. There are no effects on weight or an increased risk of hypoglycemia (in the absence of concomitant treatment with insulin or sulfonylureas) (83). The most common reported adverse events include headache, nasal pharyngitis, and upper respiratory tract infections (Table 2) (24, 84, 85). Some studies have reported a minimal increased risk of GI side effects with sitagliptin (66, 86–89). In the three head-to-head trials, there were no clinically significant differences in adverse events among DPP-4 inhibitors (90). In a population-based study using data from the United Kingdom Clinical Practice Research Datalink, use of DPP-4 inhibitors was associated with an increased risk of inflammatory bowel (91). In post-marketing reports, sitagliptin, saxagliptin, linagliptin, and alogliptin have been associated with hypersensitivity reactions, including anaphylaxis, angioedema, and more severe blistering skin conditions, including Stevens-Johnson syndrome (85). Contraindications for treatment with a DPP-4 inhibitor include serious hypersensitivity (e.g., anaphylaxis, angioedema) to a specific DPP-4 inhibitor or any component of the formulation. Some, but not all, DPP-4 inhibitors have been associated with severe joint pain (92, 93). Other reported musculoskeletal side effects include myalgias, muscle weakness, and muscle spasms. In most patients, symptoms resolved within a month after the discontinuation of the drug.

**Table 2:** Comparison of molecule type and common adverse effects of GLP-1RA vs. sitagliptin.

**DPP-4 Inhibitors**	**Molecule type**	**Common adverse effects (>5%)**
Sitagliptin	Synthetic	Nasopharyngitis, upper respiratory tract infection, peripheral edema, and headache
**GLP-1 receptor agonists (Injectable)**		
Exenatide	Synthetic, exendin-4 based, short-acting, human GLP-1 peptide	Nausea, vomiting, diarrhea, jittery feeling, dizziness, headache, dyspepsia, asthenia, gastroesophageal reflux disease, hyperhidrosis, constipation, abdominal distention, decreased appetite, and flatulence
Exenatide once-weekly	Synthetic, exendin-4 based, long-acting, human GLP-1 peptide	Nausea, diarrhea, injection-site pruritus, vomiting, injection-site nodule, constipation, headache, viral gastroenteritis, gastroesophageal reflux disease, dyspepsia, injection-site erythema, fatigue, injection-site hematoma, and decreased appetite
Lixisenatide	Synthetic, exendin-4 based, short-acting, human GLP-1 peptide	Nausea, vomiting, headache, diarrhea, dizziness, and hypoglycemia
Albiglutide	Recombinant, long acting, human GLP-1 based peptide	Diarrhea, nausea, injection site reaction, upper respiratory tract infection
Dulaglutide	Recombinant, long-acting, human GLP-1 based peptide	Nausea, diarrhea, vomiting, abdominal pain/discomfort, decreased appetite, dyspepsia, and fatigue
Semaglutide	Recombinant, long-acting, human GLP-1 based peptide	Nausea, vomiting, diarrhea, abdominal pain, and constipation
**GLP-1 receptor agonists (Oral)**		
Oral Semaglutide	Recombinant, long-acting, human GLP-1 based peptide that is coformulated with the absorption enhancer SNAC	Nausea, abdominal pain, diarrhea, vomiting, and constipation

The adverse events associated with GLP-1RA are predominantly GI, particularly nausea, vomiting, and diarrhea, and occur in 10–50% of patients in clinical trials (Table 2) (32). The risk of hypoglycemia is small. Hypoglycemic events may occur, however, when GLP-1RA are given in conjunction with diabetes medications known to cause hypoglycemia, such as basal insulin or sulfonylureas. In a meta-analysis of 17 randomized trials comparing GLP-1RA (exenatide, liraglutide, albiglutide, taspoglutide, lixisenatide) with placebo or an active comparator in patients with T2DM, patients treated with GLP-1RA experienced more nausea, diarrhea, and weight loss when compared to placebo or patients on active comparator (32). Nausea decreased with duration of therapy and was reduced with dose titration (94). GLP-1RA are associated with decreased gastric transit and should be used with caution in those with gastroparesis. In studies comparing insulin administration with once weekly GLP-1RA, including albiglutide and exenatide, local site reactions were more common with GLP-1RA (~10%), compared with 1–5% with insulin (95). Antibodies to GLP-1RA may develop, but have been shown to decreases over time without a clinical effect on glycemic control. The use of GLP-1RA is contraindicated in patients with a personal or family history of medullary thyroid carcinoma or in patients with multiple endocrine neoplasm syndrome type II (MEN−2) (90).

### Pancreatitis and Pancreatic Cancer

Since the introduction of GLP-1RA and DPP-4 inhibitors there has been some concern regarding the potential increase in pancreatitis and possibly pancreatic cancer with the incretin class of medications. Extensive, independent studies by the Food and Drug Administration which examined the potential for increased risk of pancreatitis and pancreatic cancer, showed no evidence for “pancreatic toxicity” of these two classes of medications (96). In addition, the safety data from several large cardiovascular outcome studies have demonstrated no clear increased risk of pancreatitis or pancreatic cancer (77–80). Therefore, regulatory authorities have concluded that that concerns regarding pancreatic toxicity “are inconsistent with the available scientific data” (96).

### Renal Insufficiency

Renal insufficiency is a common microvascular complication in patients with T2DM. Although the incidence of acute renal failure with the use of GLP-1RA is low there have been several reported cases in the literature (97–100). Cases of acute renal failure following treatment with DPP-4 inhibitors are rare (101). Several DPP-4 inhibitors are renally excreted including alogliptin, sitagliptin, saxagliptin and vildagliptin (90). Therefore, in patients with moderate to severe renal impairment, dosing adjustment is required. Linagliptin has a hepatic route of excretion and can be used without dose adjustment in patients at all stages of renal disease (90). Exenatide (twice daily and once weekly) and lixisenatide are renally excreted and are not recommended in patients with severe renal impairment (GFR <30 mL/min) (90). Liraglutide is not renally excreted and is approved, in the United States, for use with caution in patients at all stages of renal disease. Dulaglutide should be used with caution in patients with renal impairment.

## Summary and Recommendations

There are number of factors to consider when selecting from the many treatment options available for patients T2DM, including the multiple incretin-based therapies. Clinicians must take into account mechanisms of action, level of HbA1c reduction, impact on fasting plasma glucose and/or post-prandial glucose levels, safety and tolerability, effects on weight, route of administration, and finally, cost. GLP-1RA and DPP-4 inhibitors are useful in the management of patients with T2DM over the spectrum of HbA1c levels, including drug naïve patients as well as those treated with other glucose lowering therapies. In generally, GLP-1RA are preferred over DPP-4 inhibitors because of greater reductions in HbA1c and clinically significant weight loss observed in the clinical trials. DPP-4 inhibitors only modestly affect the levels of endogenous GLP-1, thus producing smaller glycemic reductions with minimal impact on weight reduction. Given their low risk of hypoglycemia, GLP-1RA and DPP-4 inhibitors may be preferable in patients with hypoglycemic unawareness or the elderly. In addition, several GLP-1RA have been shown to reduce a composite 3-point MACE outcome in large, randomized cardiovascular outcome trials. While the results of cardiovascular outcome trials in DPP-4 inhibitors have not shown increased risk of cardiovascular disease, they have failed to show cardiovascular benefit. Consequently, for patients with T2DM and CVD, GLP-1 RAs with a proven CV benefit are preferred in the guidelines. For obese individuals with T2DM a decrease in weight of 5–10% is clinically significant and is associated with improvements in cardiovascular risk factors (102). In many of the clinical trials comparing GLP-1RAs with sitagliptin, more than 50% of the participants treated with GLP-1RA achieved this weight loss benchmark (102). Although the perception of some clinicians is that patients may be less receptive of the GLP-1RA because they require subcutaneous injections, clinical trials have shown that patients do not associate injectable therapies with a “negative effect” as long as there is clinical efficacy (103, 104). In addition, the availability of oral semaglutide may be a potential option for patients who wish to avoid injectable therapy.

## Author Contributions

MG and RP have contributed to the conception or design of the work, participated in the drafting of the manuscript and revising it critically for important intellectual content, approved the manuscript for publication and agree to be accountable for all aspects of the work in ensuring that questions related to the accuracy or integrity of any part of the work are appropriately investigated and resolved.

### Conflict of Interest

MG has worked as a consultant for Sanofi, USA and Novo Nordisk. RP has consulted with AstraZeneca, Takeda and Novo Nordisk, Boehringer Ingelheim, GlaxoSmithKline, Hanmi Pharmaceutical Co. Ltd., Janssen Scientific Affairs LLC, Ligand Pharmaceuticals, Inc., Eli Lilly, Merck, Pfizer and Eisai, Inc. and has received research grants from Gilead Sciences, Lexicon Pharmaceuticals, Ligand Pharmaceuticals, Inc., Eli Lilly, Merck, and Takeda. Fees for his services were paid directly to AdventHealth (formerly Florida Hospital), a non-profit organization and therefore he has no financial conflict of interest with these companies.

## References

[1] Nauck MA Homberger E Siegel EG Allen RC Eaton RP Ebert R. Incretin effects of increasing glucose loads in man calculated from venous insulin and C-peptide responses. J Clin Endocrinol Metab. (1986) 63:492–8. 10.1210/jcem-63-2-492 3522621

[2] Nauck M Stockmann F Ebert R Creutzfeldt W. Reduced incretin effect in type 2 (non-insulin-dependent) diabetes. Diabetologia. (1986) 29:46–52. 10.1007/BF02427280 3514343

[3] Nauck MA Vardarli I Deacon CF Holst JJ Meier JJ. Secretion of glucagon-like peptide-1 (GLP-1) in type 2 diabetes: what is up, what is down? Diabetologia. (2011) 54:10–8. 10.1007/s00125-010-1896-4 20871975

[4] Calanna S Christensen M Holst JJ Laferrere B Gluud LL Vilsboll T. Secretion of glucagon-like peptide-1 in patients with type 2 diabetes mellitus: systematic review and meta-analyses of clinical studies. Diabetologia. (2013) 56:965–72. 10.1007/s00125-013-2841-0 23377698 PMC3687347

[5] Adrian TE Ferri GL Bacarese-Hamilton AJ Fuessl HS Polak JM Bloom SR. Human distribution and release of a putative new gut hormone, peptide YY. Gastroenterology. (1985) 89:1070–7. 10.1016/0016-5085(85)90211-2 3840109

[6] Vilsboll T Holst JJ. Incretins, insulin secretion and Type 2 diabetes mellitus. Diabetologia. (2004) 47:357–66. 10.1007/s00125-004-1342-6 14968296

[7] Drucker DJ Nauck MA. The incretin system: glucagon-like peptide-1 receptor agonists and dipeptidyl peptidase-4 inhibitors in type 2 diabetes. Lancet. (2006) 368:1696–705. 10.1016/S0140-6736(06)69705-5 17098089

[8] Komatsu R Matsuyama T Namba M Watanabe N Itoh H Kono N. Glucagonostatic and insulinotropic action of glucagonlike peptide I-(7-36)-amide. Diabetes. (1989) 38:902–5. 10.2337/diab.38.7.902 2661287

[9] Nauck MA Kleine N Orskov C Holst JJ Willms B Creutzfeldt W. Normalization of fasting hyperglycaemia by exogenous glucagon-like peptide 1 (7-36 amide) in type 2 (non-insulin-dependent) diabetic patients. Diabetologia. (1993) 36:741–4. 10.1007/BF00401145 8405741

[10] Willms B Werner J Holst JJ Orskov C Creutzfeldt W Nauck MA. Gastric emptying, glucose responses, and insulin secretion after a liquid test meal: effects of exogenous glucagon-like peptide-1 (GLP-1)-(7-36) amide in type 2 (noninsulin-dependent) diabetic patients. J Clin Endocrinol Metab. (1996) 81:327–32. 10.1210/jcem.81.1.8550773 8550773

[11] Naslund E Gutniak M Skogar S Rossner S Hellstrom PM. Glucagon-like peptide 1 increases the period of postprandial satiety and slows gastric emptying in obese men. Am J Clin Nutr. (1998) 68:525–30. 10.1093/ajcn/68.3.525 9734726

[12] Flint A Raben A Ersboll AK Holst JJ Astrup A. The effect of physiological levels of glucagon-like peptide-1 on appetite, gastric emptying, energy and substrate metabolism in obesity. Int J Obes Relat Metab Disord. (2001) 25:781–92. 10.1038/sj.ijo.0801627 11439290

[13] Nauck MA Heimesaat MM Behle K Holst JJ Nauck MS Ritzel R. Effects of glucagon-like peptide 1 on counterregulatory hormone responses, cognitive functions, and insulin secretion during hyperinsulinemic, stepped hypoglycemic clamp experiments in healthy volunteers. J Clin Endocrinol Metab. (2002) 87:1239–46. 10.1210/jcem.87.3.8355 11889194

[14] Holst JJ Gromada J. Role of incretin hormones in the regulation of insulin secretion in diabetic and nondiabetic humans. Am J Physiol Endocrinol Metab. (2004) 287:E199–206. 10.1152/ajpendo.00545.2003 15271645

[15] Hare KJ Vilsboll T Asmar M Deacon CF Knop FK Holst JJ. The glucagonostatic and insulinotropic effects of glucagon-like peptide 1 contribute equally to its glucose-lowering action. Diabetes. (2010) 59:1765–70. 10.2337/db09-1414 20150286 PMC2889777

[16] Nauck MA Niedereichholz U Ettler R Holst JJ Orskov C Ritzel R. Glucagon-like peptide 1 inhibition of gastric emptying outweighs its insulinotropic effects in healthy humans. Am J Physiol. (1997) 273(5 Pt 1):E981–8. 10.1152/ajpendo.1997.273.5.E981 9374685

[17] Vilsboll T Krarup T Madsbad S Holst JJ. Defective amplification of the late phase insulin response to glucose by GIP in obese Type II diabetic patients. Diabetologia. (2002) 45:1111–9. 10.1007/s00125-002-0878-6 12189441

[18] Mentis N Vardarli I Kothe LD Holst JJ Deacon CF Theodorakis M GIP does not potentiate the antidiabetic effects of GLP-1 in hyperglycemic patients with type 2 diabetes. Diabetes. (2011) 60:1270–6. 10.2337/db10-1332 21330636 PMC3064100

[19] Mentlein R Gallwitz B Schmidt WE. Dipeptidyl-peptidase IV hydrolyses gastric inhibitory polypeptide, glucagon-like peptide-1(7-36)amide, peptide histidine methionine and is responsible for their degradation in human serum. Eur J Biochem. (1993) 214:829–35. 10.1111/j.1432-1033.1993.tb17986.x 8100523

[20] Vahl TP Paty BW Fuller BD Prigeon RL D'Alessio DA. Effects of GLP-1-(7-36)NH2, GLP-1-(7-37), and GLP-1- (9-36)NH2 on intravenous glucose tolerance and glucose-induced insulin secretion in healthy humans. J Clin Endocrinol Metab. (2003) 88:1772–9. 10.1210/jc.2002-021479 12679472

[21] Demuth HU McIntosh CH Pederson RA. Type 2 diabetes–therapy with dipeptidyl peptidase IV inhibitors. Biochim Biophys Acta. (2005) 1751:33–44. 10.1016/j.bbapap.2005.05.010 15978877

[22] Craddy P Palin HJ Johnson KI. Comparative effectiveness of dipeptidylpeptidase-4 inhibitors in type 2 diabetes: a systematic review and mixed treatment comparison. Diabetes Ther. (2014) 5:1–41. 10.1007/s13300-014-0061-3 24664619 PMC4065303

[23] Scheen AJ Charpentier G Ostgren CJ Hellqvist A Gause-Nilsson I. Efficacy and safety of saxagliptin in combination with metformin compared with sitagliptin in combination with metformin in adult patients with type 2 diabetes mellitus. Diabetes Metab Res Rev. (2010) 26:540–9. 10.1002/dmrr.1114 20824678

[24] Amori RE Lau J Pittas AG. Efficacy and safety of incretin therapy in type 2 diabetes: systematic review and meta-analysis. JAMA. (2007) 298:194–206. 10.1001/jama.298.2.194 17622601

[25] Madsbad S. Exenatide and liraglutide: different approaches to develop GLP-1 receptor agonists (incretin mimetics)–preclinical and clinical results. Best Pract Res Clin Endocrinol Metab. (2009) 23:463–77. 10.1016/j.beem.2009.03.008 19748064

[26] Gerich J. DPP-4 inhibitors: what may be the clinical differentiators? Diabetes Res Clin Pract. (2010) 90:131–40. 10.1016/j.diabres.2010.07.006 20708812

[27] Stonehouse A Walsh B Cuddihy R. Exenatide once-weekly clinical development: safety and efficacy across a range of background therapies. Diabetes Technol Ther. (2011) 13:1063–9. 10.1089/dia.2011.0076 21732798 PMC3303092

[28] Holst JJ. The physiology of glucagon-like peptide 1. Physiol Rev. (2007) 87:1409–39. 10.1152/physrev.00034.2006 17928588

[29] Meier JJ. GLP-1 receptor agonists for individualized treatment of type 2 diabetes mellitus. Nat Rev Endocrinol. (2012) 8:728–42. 10.1038/nrendo.2012.140 22945360

[30] Drucker DJ. Mechanisms of action and therapeutic application of glucagon-like peptide-1. Cell Metab. (2018) 27:740–56. 10.1016/j.cmet.2018.03.001 29617641

[31] Uccellatore A Genovese S Dicembrini I Mannucci E Ceriello A. Comparison review of short-acting and long-acting glucagon-like peptide-1 receptor agonists. Diabetes Ther. (2015) 6:239–56. 10.1007/s13300-015-0127-x 26271795 PMC4575308

[32] Shyangdan DS Royle P Clar C Sharma P Waugh N Snaith A. Glucagon-like peptide analogues for type 2 diabetes mellitus. Cochrane Database Syst Rev. (2011) 10:CD006423. 10.1002/14651858.CD006423.pub2 21975753 PMC6486297

[33] Buse JB Henry RR Han J Kim DD Fineman MS Baron AD. Effects of exenatide (exendin-4) on glycemic control over 30 weeks in sulfonylurea-treated patients with type 2 diabetes. Diabetes Care. (2004) 27:2628–35. 10.2337/diacare.27.11.2628 15504997

[34] Hermansen K Kipnes M Luo E Fanurik D Khatami H Stein P Efficacy and safety of the dipeptidyl peptidase-4 inhibitor, sitagliptin, in patients with type 2 diabetes mellitus inadequately controlled on glimepiride alone or on glimepiride and metformin. Diabetes Obes Metab. (2007) 9:733–45. 10.1111/j.1463-1326.2007.00744.x 17593236

[35] Davidson JA. Advances in therapy for type 2 diabetes: GLP-1 receptor agonists and DPP-4 inhibitors. Cleve Clin J Med. (2009) 76 (Suppl) 5:S28–38. 10.3949/ccjm.76.s5.05 19952301

[36] Marre M Shaw J Brandle M Bebakar WMW Kamaruddin NA Strand J. Liraglutide, a once-daily human GLP-1 analogue, added to a sulphonylurea over 26 weeks produces greater improvements in glycaemic and weight control compared with adding rosiglitazone or placebo in subjects with Type 2 diabetes (LEAD-1 SU). Diabetes Med. (2009) 26:268–78. 10.1111/j.1464-5491.2009.02666.x 19317822 PMC2871176

[37] Diamant M Van Gaal L Stranks S Northrup J Cao D Taylor K. Once weekly exenatide compared with insulin glargine titrated to target in patients with type 2 diabetes (DURATION-3): an open-label randomised trial. Lancet. (2010) 375:2234–43. 10.1016/S0140-6736(10)60406-0 20609969

[38] Blevins T Pullman J Malloy J Yan P Taylor K Schulteis C. DURATION-5: exenatide once weekly resulted in greater improvements in glycemic control compared with exenatide twice daily in patients with type 2 diabetes. J Clin Endocrinol Metab. (2011) 96:1301–10. 10.1210/jc.2010-2081 21307137

[39] Wysham C Blevins T Arakaki R Colon G Garcia P Atisso C. Efficacy and safety of dulaglutide added onto pioglitazone and metformin versus exenatide in type 2 diabetes in a randomized controlled trial (AWARD-1). Diabetes Care. (2014) 37:2159–67. 10.2337/dc13-2760 24879836

[40] Ahmann AJ Capehorn M Charpentier G Dotta F Henkel E Lingvay I. Efficacy and safety of once-weekly semaglutide versus exenatide er in subjects with type 2 diabetes (SUSTAIN 3): A 56-week, open-label, randomized clinical trial. Diabetes Care. (2018) 41:258–66. 10.2337/dc17-0417 29246950

[41] Pratley RE Aroda VR Lingvay I Ludemann J Andreassen C Navarria A. Semaglutide versus dulaglutide once weekly in patients with type 2 diabetes (SUSTAIN 7): a randomised, open-label, phase 3b trial. Lancet Diabetes Endocrinol. (2018) 6:275–86. 10.1016/S2213-8587(18)30024-X 29397376

[42] DeFronzo RA Okerson T Viswanathan P Guan X Holcombe JH MacConell L. Effects of exenatide versus sitagliptin on postprandial glucose, insulin and glucagon secretion, gastric emptying, and caloric intake: a randomized, cross-over study. Curr Med Res Opin. (2008) 24:2943–52. 10.1185/03007990802418851 18786299

[43] Berg JK Shenouda SK Heilmann CR Gray AL Holcombe JH. Effects of exenatide twice daily versus sitagliptin on 24-h glucose, glucoregulatory and hormonal measures: a randomized, double-blind, crossover study. Diabetes Obes Metab. (2011) 13:982–9. 10.1111/j.1463-1326.2011.01428.x 21615670 PMC3258427

[44] Bergenstal RM Wysham C Macconell L Malloy J Walsh B Yan P. Efficacy and safety of exenatide once weekly versus sitagliptin or pioglitazone as an adjunct to metformin for treatment of type 2 diabetes (DURATION-2): a randomised trial. Lancet. (2010) 376:431–9. 10.1016/S0140-6736(10)60590-9 20580422

[45] Pratley RE Nauck M Bailey T Montanya E Cuddihy R Filetti S Liraglutide versus sitagliptin for patients with type 2 diabetes who did not have adequate glycaemic control with metformin: a 26-week, randomised, parallel-group, open-label trial. Lancet. (2010) 375:1447–56. 10.1016/S0140-6736(10)60307-8 20417856

[46] Van Gaal L Souhami E Zhou T Aronson R. Efficacy and safety of the glucagon-like peptide-1 receptor agonist lixisenatide versus the dipeptidyl peptidase-4 inhibitor sitagliptin in young (<50 years) obese patients with type 2 diabetes mellitus. J Clin Transl Endocrinol. (2014) 1:31–7. 10.1016/j.jcte.2014.03.001 29159080 PMC5685032

[47] Bergenstal RM Forti A Chiasson JL Woloschak M Boldrin M Balena R. Efficacy and safety of taspoglutide versus sitagliptin for type 2 diabetes mellitus (T-emerge 4 trial). Diabetes Ther. (2012). 3:13. 10.1007/s13300-012-0013-8. 23138449 PMC3508113

[48] Ahren B Johnson SL Stewart M Cirkel DT Yang F Perry C. HARMONY 3: 104-week randomized, double-blind, placebo- and active-controlled trial assessing the efficacy and safety of albiglutide compared with placebo, sitagliptin, and glimepiride in patients with type 2 diabetes taking metformin. Diabetes Care. (2014) 37:2141–8. 10.2337/dc14-0024 24898304

[49] Leiter LA Carr MC Stewart M Jones-Leone A Scott R Yang F. Efficacy and safety of the once-weekly GLP-1 receptor agonist albiglutide versus sitagliptin in patients with type 2 diabetes and renal impairment: a randomized phase III study. Diabetes Care. (2014) 37:2723–30. 10.2337/dc13-2855 25048383

[50] Nauck M Weinstock RS Umpierrez GE Guerci B Skrivanek Z Milicevic Z. Efficacy and safety of dulaglutide versus sitagliptin after 52 weeks in type 2 diabetes in a randomized controlled trial (AWARD-5). Diabetes Care. (2014) 37:2149–58. 10.2337/dc13-2761 24742660 PMC4113177

[51] Weinstock RS Guerci B Umpierrez G Nauck MA Skrivanek Z Milicevic Z. Safety and efficacy of once-weekly dulaglutide versus sitagliptin after 2 years in metformin-treated patients with type 2 diabetes (AWARD-5): a randomized, phase III study. Diabetes Obes Metab. (2015) 17:849–58. 10.1111/dom.12479 25912221 PMC5008205

[52] Ahren B Masmiquel L Kumar H Sargin M Karsbol JD Jacobsen SH. Efficacy and safety of once-weekly semaglutide versus once-daily sitagliptin as an add-on to metformin, thiazolidinediones, or both, in patients with type 2 diabetes (SUSTAIN 2): a 56-week, double-blind, phase 3a, randomised trial. Lancet Diabetes Endocrinol. (2017) 5:341–54. 10.1016/S2213-8587(17)30092-X 28385659

[53] Seino Y Min KW Niemoeller E Takami A Investigators EG-LAS Randomized, double-blind, placebo-controlled trial of the once-daily GLP-1 receptor agonist lixisenatide in Asian patients with type 2 diabetes insufficiently controlled on basal insulin with or without a sulfonylurea (GetGoal-L-Asia). Diabetes Obes Metab. (2012) 14:910–7. 10.1111/j.1463-1326.2012.01618.x 22564709 PMC3466411

[54] Rosenstock J Allison D Birkenfeld AL Blicher TM Deenadayalan S Jacobsen JB. Effect of additional oral semaglutide vs sitagliptin on glycated hemoglobin in adults with type 2 diabetes uncontrolled with metformin alone or with sulfonylurea: the PIONEER 3 randomized clinical trial. JAMA. (2019) 321:1466–80. 10.1001/jama.2019.2942 30903796 PMC6484814

[55] Kolterman OG Buse JB Fineman MS Gaines E Heintz S Bicsak TA. Synthetic exendin-4 (exenatide) significantly reduces postprandial and fasting plasma glucose in subjects with type 2 diabetes. J Clin Endocrinol Metab. (2003) 88:3082–9. 10.1210/jc.2002-021545 12843147

[56] Herman GA Stevens C Van Dyck K Bergman A Yi B De Smet M. Pharmacokinetics and pharmacodynamics of sitagliptin, an inhibitor of dipeptidyl peptidase IV, in healthy subjects: results from two randomized, double-blind, placebo-controlled studies with single oral doses. Clin Pharmacol Ther. (2005) 78:675–88. 10.1016/j.clpt.2005.09.002 16338283

[57] Elbrond B Jakobsen G Larsen S Agerso H Jensen LB Rolan P. Pharmacokinetics, pharmacodynamics, safety, and tolerability of a single-dose of NN2211, a long-acting glucagon-like peptide 1 derivative, in healthy male subjects. Diabetes Care. (2002) 25:1398–404. 10.2337/diacare.25.8.1398 12145241

[58] Matthews JE Stewart MW De Boever EH Dobbins RL Hodge RJ Walker SE. Pharmacodynamics, pharmacokinetics, safety, and tolerability of albiglutide, a long-acting glucagon-like peptide-1 mimetic, in patients with type 2 diabetes. J Clin Endocrinol Metab. (2008) 93:4810–7. 10.1210/jc.2008-1518 18812476

[59] Rosenstock J Reusch J Bush M Yang F Stewart M Albiglutide Study G. Potential of albiglutide, a long-acting GLP-1 receptor agonist, in type 2 diabetes: a randomized controlled trial exploring weekly, biweekly, and monthly dosing. Diabetes Care. (2009) 32:1880–6. 10.2337/dc09-0366 19592625 PMC2752910

[60] Barrington P Chien JY Tibaldi F Showalter HD Schneck K Ellis B. LY2189265, a long-acting glucagon-like peptide-1 analogue, showed a dose-dependent effect on insulin secretion in healthy subjects. Diabetes Obes Metab. (2011) 13:434–8. 10.1111/j.1463-1326.2011.01365.x 21251179

[61] Lau J Bloch P Schaffer L Pettersson I Spetzler J Kofoed J. Discovery of the Once-Weekly Glucagon-Like Peptide-1 (GLP-1) Analogue Semaglutide. J Med Chem. (2015) 58:7370–80. 10.1021/acs.jmedchem.5b00726 26308095

[62] Philippart M Schmidt J Bittner B. Oral delivery of therapeutic proteins and peptides: an overview of current technologies and recommendations for bridging from approved intravenous or subcutaneous administration to novel oral regimens. Drug Res. (2016) 66:113–20. 10.1055/s-0035-1559654 26536331

[63] Davies M Pieber TR Hartoft-Nielsen ML Hansen OKH Jabbour S Rosenstock J. Effect of oral semaglutide compared with placebo and subcutaneous semaglutide on glycemic control in patients with type 2 diabetes: a randomized clinical trial. JAMA. (2017) 318:1460–70. 10.1001/jama.2017.14752 29049653 PMC5817971

[64] Pieber TR Bode B Mertens A Cho YM Christiansen E Hertz CL. Efficacy and safety of oral semaglutide with flexible dose adjustment versus sitagliptin in type 2 diabetes (PIONEER 7): a multicentre, open-label, randomised, phase 3a trial. Lancet Diabetes Endocrinol. (2019) 7:528–39. 10.1016/S2213-8587(19)30194-9 31189520

[65] Degn KB Juhl CB Sturis J Jakobsen G Brock B Chandramouli V. One week's treatment with the long-acting glucagon-like peptide 1 derivative liraglutide (NN2211) markedly improves 24-h glycemia and alpha- and beta-cell function and reduces endogenous glucose release in patients with type 2 diabetes. Diabetes. (2004) 53:1187–94. 10.2337/diabetes.53.5.1187 15111485

[66] Aschner P Kipnes MS Lunceford JK Sanchez M Mickel C Williams-Herman DE. Effect of the dipeptidyl peptidase-4 inhibitor sitagliptin as monotherapy on glycemic control in patients with type 2 diabetes. Diabetes Care. (2006) 29:2632–7. 10.2337/dc06-0703 17130196

[67] McGill JB. Insights from the liraglutide clinical development program–the liraglutide effect and action in diabetes (LEAD) studies. Postgrad Med. (2009) 121:16–25. 10.3810/pgm.2009.05.1998 19491536

[68] Wysham C Bergenstal R Malloy J Yan P Walsh B Malone J DURATION-2: efficacy and safety of switching from maximum daily sitagliptin or pioglitazone to once-weekly exenatide. Diabet Med. (2011) 28:705–14. 10.1111/j.1464-5491.2011.03301.x 21434995 PMC3123706

[69] Pratley RE Nauck MA Bailey T Montanya E Filetti S Garber AJ. Efficacy and safety of switching from the DPP-4 inhibitor sitagliptin to the human GLP-1 analog liraglutide after 52 weeks in metformin-treated patients with type 2 diabetes: a randomized, open-label trial. Diabetes Care. (2012) 35:1986–93. 10.2337/dc11-2113 22851600 PMC3447855

[70] Bailey TS Takacs R Tinahones FJ Rao PV Tsoukas GM Thomsen AB. Efficacy and safety of switching from sitagliptin to liraglutide in subjects with type 2 diabetes (LIRA-SWITCH): a randomized, double-blind, double-dummy, active-controlled 26-week trial. Diabetes Obes Metab. (2016) 18:1191–8. 10.1111/dom.12736 27381275 PMC5129465

[71] Violante R Oliveira JH Yoon KH Reed VA Yu MB Bachmann OP. A randomized non-inferiority study comparing the addition of exenatide twice daily to sitagliptin or switching from sitagliptin to exenatide twice daily in patients with type 2 diabetes experiencing inadequate glycaemic control on metformin and sitagliptin. Diabet Med. (2012). 29:e417–24. 10.1111/j.1464-5491.2012.03624.x. 22375612

[72] Nauck MA Kahle M Baranov O Deacon CF Holst JJ. Addition of a dipeptidyl peptidase-4 inhibitor, sitagliptin, to ongoing therapy with the glucagon-like peptide-1 receptor agonist liraglutide: A randomized controlled trial in patients with type 2 diabetes. Diabetes Obes Metab. (2017). 19:200–207. 10.1111/dom.12802. 27709794

[73] Scirica BM Bhatt DL Braunwald E Steg PG Davidson J Hirshberg B. Saxagliptin and cardiovascular outcomes in patients with type 2 diabetes mellitus. N Engl J Med. (2013) 369:1317–26. 10.1056/NEJMoa1307684 23992601

[74] White WB Cannon CP Heller SR Nissen SE Bergenstal RM Bakris GL. Alogliptin after acute coronary syndrome in patients with type 2 diabetes. N Engl J Med. (2013) 369:1327–35. 10.1056/NEJMoa1305889 23992602

[75] Green JB Bethel MA Armstrong PW Buse JB Engel SS Garg J. Effect of sitagliptin on cardiovascular outcomes in type 2 diabetes. N Engl J Med. (2015) 373:232–42. 10.1056/NEJMoa1501352 26052984

[76] Cefalu WT Kaul S Gerstein HC Holman RR Zinman B Skyler JS. Cardiovascular outcomes trials in type 2 diabetes: where do we go from here? Reflections From a Diabetes Care Editors' Expert Forum. Diabetes Care. (2018) 41:14–31. 10.2337/dci17-0057 29263194 PMC5741160

[77] Pfeffer MA Claggett B Diaz R Dickstein K Gerstein HC Kober LV. Lixisenatide in patients with type 2 diabetes and acute Coronary syndrome. N Engl J Med. (2015) 373:2247–57. 10.1056/NEJMoa1509225 26630143

[78] Marso SP Daniels GH Brown-Frandsen K Kristensen P Mann JF Nauck MA. Liraglutide and cardiovascular outcomes in type 2 diabetes. N Engl J Med. (2016) 375:311–22. 10.1056/NEJMoa1603827 27295427 PMC4985288

[79] Marso SP Bain SC Consoli A Eliaschewitz FG Jodar E Leiter LA. Semaglutide and cardiovascular outcomes in patients with type 2 diabetes. N Engl J Med. (2016) 375:1834–44. 10.1056/NEJMoa1607141 27633186

[80] Holman RR Bethel MA Mentz RJ Thompson VP Lokhnygina Y Buse JB. Effects of once-weekly exenatide on cardiovascular outcomes in type 2 diabetes. N Engl J Med. (2017) 377:1228–39. 10.1056/NEJMoa1612917 28910237 PMC9792409

[81] Gerstein HC Colhoun HM Dagenais GR Diaz R Lakshmanan M Pais P. Dulaglutide and cardiovascular outcomes in type 2 diabetes (REWIND): a double-blind, randomised placebo-controlled trial. Lancet. (2019) 394:121–30. 10.1016/S0140-6736(19)31149-3 31189511

[82] Husain M Birkenfeld AL Donsmark M Dungan K Eliaschewitz FG Franco DR. Oral semaglutide and cardiovascular outcomes in patients with type 2 diabetes. N Engl J Med. (2019) 381:841–51. 10.1056/NEJMoa1901118 31185157

[83] Salvo F Moore N Arnaud M Robinson P Raschi E De Ponti F. Addition of dipeptidyl peptidase-4 inhibitors to sulphonylureas and risk of hypoglycaemia: systematic review and meta-analysis. BMJ. (2016) 353:i2231. 10.1136/bmj.i2231 27142267 PMC4854021

[84] Del Prato S Barnett AH Huisman H Neubacher D Woerle HJ Dugi KA. Effect of linagliptin monotherapy on glycaemic control and markers of beta-cell function in patients with inadequately controlled type 2 diabetes: a randomized controlled trial. Diabetes Obes Metab. (2011) 13:258–67. 10.1111/j.1463-1326.2010.01350.x 21205122

[85] Goossen K Graber S. Longer term safety of dipeptidyl peptidase-4 inhibitors in patients with type 2 diabetes mellitus: systematic review and meta-analysis. Diabetes Obes Metab. (2012) 14:1061–72. 10.1111/j.1463-1326.2012.01610.x 22519906

[86] Charbonnel B Karasik A Liu J Wu M Meininger G Sitagliptin Study G. Efficacy and safety of the dipeptidyl peptidase-4 inhibitor sitagliptin added to ongoing metformin therapy in patients with type 2 diabetes inadequately controlled with metformin alone. Diabetes Care. (2006) 29:2638–43. 10.2337/dc06-0706 17130197

[87] Raz I Hanefeld M Xu L Caria C Williams-Herman D Khatami H. Efficacy and safety of the dipeptidyl peptidase-4 inhibitor sitagliptin as monotherapy in patients with type 2 diabetes mellitus. Diabetologia. (2006) 49:2564–71. 10.1007/s00125-006-0416-z 17001471

[88] Rosenstock J Brazg R Andryuk PJ Lu K Stein P Sitagliptin Study G. Efficacy and safety of the dipeptidyl peptidase-4 inhibitor sitagliptin added to ongoing pioglitazone therapy in patients with type 2 diabetes: a 24-week, multicenter, randomized, double-blind, placebo-controlled, parallel-group study. Clin Ther. (2006) 28:1556–68. 10.1016/j.clinthera.2006.10.007 17157112

[89] Bosi E Camisasca RP Collober C Rochotte E Garber AJ. Effects of vildagliptin on glucose control over 24 weeks in patients with type 2 diabetes inadequately controlled with metformin. Diabetes Care. (2007) 30:890–5. 10.2337/dc06-1732 17277036

[90] Brunton S. GLP-1 receptor agonists vs. DPP-4 inhibitors for type 2 diabetes: is one approach more successful or preferable than the other? Int J Clin Pract. (2014) 68:557–67. 10.1111/ijcp.12361 24499291 PMC4238422

[91] Abrahami D Douros A Yin H Yu OHY Renoux C Bitton A. Dipeptidyl peptidase-4 inhibitors and incidence of inflammatory bowel disease among patients with type 2 diabetes: population based cohort study. BMJ. (2018) 360:k872. 10.1136/bmj.k872 29563098 PMC5861502

[92] Chaicha-Brom T Yasmeen T. DPP-IV inhibitor-associated arthralgias. Endocr Pract. (2013) 19:377. 10.4158/EP13076.LT 23598537

[93] Tarapues M Cereza G Figueras A. Association of musculoskeletal complaints and gliptin use: review of spontaneous reports. Pharmacoepidemiol Drug Saf. (2013) 22:1115–8. 10.1002/pds.3503 23960039

[94] Fineman MS Shen LZ Taylor K Kim DD Baron AD. Effectiveness of progressive dose-escalation of exenatide (exendin-4) in reducing dose-limiting side effects in subjects with type 2 diabetes. Diabetes Metab Res Rev. (2004) 20:411–7. 10.1002/dmrr.499 15343588

[95] Rosenstock J Fonseca VA Gross JL Ratner RE Ahren B Chow FC. Advancing basal insulin replacement in type 2 diabetes inadequately controlled with insulin glargine plus oral agents: a comparison of adding albiglutide, a weekly GLP-1 receptor agonist, versus thrice-daily prandial insulin lispro. Diabetes Care. (2014) 37:2317–25. 10.2337/dc14-0001 24898300

[96] Egan AG Blind E Dunder K de Graeff PA Hummer BT Bourcier T. Pancreatic safety of incretin-based drugs–FDA and EMA assessment. N Engl J Med. (2014) 370:794–7. 10.1056/NEJMp1314078 24571751

[97] Weise WJ Sivanandy MS Block CA Comi RJ. Exenatide-associated ischemic renal failure. Diabetes Care. (2009) 32:e22–23. 10.2337/dc08-1309 19171732 PMC6908415

[98] Lopez-Ruiz A del Peso-Gilsanz C Meoro-Aviles A Soriano-Palao J Andreu A Cabezuelo J. Acute renal failure when exenatide is co-administered with diuretics and angiotensin II blockers. Pharm World Sci. (2010) 32:559–61. 10.1007/s11096-010-9423-8 20686848

[99] Kaakeh Y Kanjee S Boone K Sutton J. Liraglutide-induced acute kidney injury. Pharmacotherapy. (2012) 32:e7–11. 10.1002/PHAR.1014 22392833

[100] Nandakoban H Furlong TJ Flack JR Acute tubulointerstitial nephritis following treatment with exenatide. Diabet Med. (2013) 30:123–5. 10.1111/j.1464-5491.2012.03738.x 22762797

[101] Leibovitz E Gottlieb S Goldenberg I Gevrielov-Yusim N Matetzky S Gavish D. Sitagliptin pretreatment in diabetes patients presenting with acute coronary syndrome: results from the Acute Coronary Syndrome Israeli Survey (ACSIS). Cardiovasc Diabetol. (2013) 12:53. 10.1186/1475-2840-12-53 23537430 PMC3637090

[102] Wing RR Lang W Wadden TA Safford M Knowler WC Bertoni AG. Benefits of modest weight loss in improving cardiovascular risk factors in overweight and obese individuals with type 2 diabetes. Diabetes Care. (2011) 34:1481–6. 10.2337/dc10-2415 21593294 PMC3120182

[103] Nicolucci A Cucinotta D Squatrito S Lapolla A Musacchio N Leotta S. Clinical and socio-economic correlates of quality of life and treatment satisfaction in patients with type 2 diabetes. Nutr Metab Cardiovasc Dis. (2009) 19:45–53. 10.1016/j.numecd.2007.12.005 18450436

[104] Pratley R Nauck M Bailey T Montanya E Cuddihy R Filetti S. One year of liraglutide treatment offers sustained and more effective glycaemic control and weight reduction compared with sitagliptin, both in combination with metformin, in patients with type 2 diabetes: a randomised, parallel-group, open-label trial. Int J Clin Pract. (2011) 65:397–407. 10.1111/j.1742-1241.2011.02656.x 21355967 PMC3085127

